# Oleate ameliorates palmitate-induced reduction of NAMPT activity and NAD levels in primary human hepatocytes and hepatocarcinoma cells

**DOI:** 10.1186/s12944-017-0583-6

**Published:** 2017-10-03

**Authors:** Melanie Penke, Susanne Schuster, Theresa Gorski, Rolf Gebhardt, Wieland Kiess, Antje Garten

**Affiliations:** 1 Center for Pediatric Research Leipzig (CPL), University Hospital for Children & Adolescents, University of Leipzig, Liebigstraße 21, 04103 Leipzig, Germany; 2 Institute of Biochemistry, Faculty of Medicine, University of Leipzig, Johannisallee 30, 04103 Leipzig, Germany

**Keywords:** NAMPT, NAD, NMN, FK866, Steatosis, Liver, Palmitate, Oleate

## Abstract

**Background:**

Nicotinamide phosphoribosyltransferase (NAMPT) and nicotinamide adenine dinucleotide (NAD) levels are crucial for liver function. The saturated fatty acid palmitate and the unsaturated fatty acid oleate are the main free fatty acids in adipose tissue and human diet. We asked how these fatty acids affect cell survival, NAMPT and NAD levels in HepG2 cells and primary human hepatocytes.

**Methods:**

HepG2 cells were stimulated with palmitate (0.5mM), oleate (1mM) or a combination of both (0.5mM/1mM) as well as nicotinamide mononucleotide (NMN) (0.5 mM) or the specific NAMPT inhibitor FK866 (10nM). Cell survival was measured by WST-1 assay and Annexin V/propidium iodide staining. NAD levels were determined by NAD/NADH Assay or HPLC. Protein and mRNA levels were analysed by Western blot analyses and qPCR, respectively. NAMPT enzyme activity was measured using radiolabelled ^14^C–nicotinamide. Lipids were stained by Oil red O staining.

**Results:**

Palmitate significantly reduced cell survival and induced apoptosis at physiological doses. NAMPT activity and NAD levels significantly declined after 48h of palmitate. In addition, *NAMPT* mRNA expression was enhanced which was associated with increased NAMPT release into the supernatant, while intracellular NAMPT protein levels remained stable. Oleate alone did not influence cell viability and NAMPT activity but ameliorated the negative impact of palmitate on cell survival, NAMPT activity and NAD levels, as well as the increased *NAMPT* mRNA expression and secretion. NMN was able to normalize intracellular NAD levels but did not ameliorate cell viability after co-stimulation with palmitate. FK866, a specific NAMPT inhibitor did not influence lipid accumulation after oleate-treatment.

**Conclusions:**

Palmitate targets NAMPT activity with a consequent cellular depletion of NAD. Oleate protects from palmitate-induced apoptosis and variation of NAMPT and NAD levels. Palmitate-induced cell stress leads to an increase of NAMPT mRNA and accumulation in the supernatant. However, the proapoptotic action of palmitate seems not to be mediated by decreased NAD levels.

**Electronic supplementary material:**

The online version of this article (10.1186/s12944-017-0583-6) contains supplementary material, which is available to authorized users.

## Background

Nicotinamide phosphoribosyltransferase (NAMPT, also called visfatin or pre-B cell colony-enhancing factor (PBEF)) is a key enzyme of mammalian nicotinamide adenine dinucleotide (NAD) biosynthesis converting nicotinamide to nicotinamide mononucleotide (NMN) [1, 2]. By regulating intracellular NAD levels and the activity of NAD dependent enzymes, for example sirtuins, NAMPT is involved in the regulation of cellular metabolism [2]. Moreover, NAMPT was shown to be differentially regulated in pathophysiological conditions like cancer [3], inflammatory disorders [4], heart diseases [5] and metabolic disorders, such as type 2 diabetes [6], obesity [7] and non-alcoholic fatty liver (NAFLD) [8–11]. Hepatic steatosis develops when triglycerides are accumulating in the liver due to insulin resistance, as a consequence of disturbed lipolysis in adipose tissue and de novo lipogenesis in the liver [12]. An improvement of NAMPT protein and NAD levels by the bioflavonoid troxerutin or leucine was shown to protect mice with diet-induced obesity against steatosis, inflammation and glucose intolerance [13, 14] indicating an important role of NAMPT in the development of NAFLD. However, less is known about the regulation of hepatic NAMPT by the two main free fatty acids of human nutrition and adipose tissue, the monounsaturated fatty acid oleate (18:1) and the saturated fatty acid palmitate (16:0) [15, 16]. In this study, we investigated the impact of palmitate and oleate on NAMPT-mediated NAD biosynthesis in HepG2 cells and primary human hepatocytes. We could show that palmitate down regulated NAMPT activity and NAD levels while oleate ameliorated the negative effect of palmitate. However, hepatic NAD levels seemed to have no effect on hepatocyte survival or fat accumulation.

## Methods

### Cell culture

HepG2 cells were purchased from Leibniz Institute DSMZ (German Collection of Microorganisms and Cell Cultures) and cultured in MEM (Life Technologies) containing 10% fetal calf serum, 1% L-glutamine and 1% of streptomycin and penicillin (PAA Laboratories GmbH) at 37 °C in a humidified atmosphere of 5% CO_2_. Cells were passaged every 2 to 3 days. For experiments, cells were seeded in serum containing medium overnight. After washing with PBS, cells were serum starved for 24 h. Primary human hepatocytes were isolated as described before [17] and seeded in Williams’ Medium E containing 10% fetal calf serum, 1% L-glutamine and 1% of streptomycin and penicillin as well as 10–7 mol/L dexamethansone. Afterwards, cells were incubated with palmitate (0.5 mM), oleate (1.0 mM) or a combination of palmitate and oleate (0.5 mM/1 mM), dissolved as described previously, for 4 h, 24 h and 48 h, as well as nicotinamide mononucleotide (NMN; 0.5 mM) and FK866 (10 nM, Sigma-Aldrich). NMN was dissolved in serum free medium, FK866 in DMSO (dilution factor 1:1′000‘000) and palmitate and oleate in NaOH (dilution factor 1:200). All solvent controls had no impact on cell viability, NAMPT mRNA expression, protein levels and NAMPT activity as well as lipid accumulation (Additional file 1: Figure S1).

### Cell viability assay

Cell viability was measured by using the Cell Proliferation Reagent WST-1 (Roche Diagnostics GmbH) according to manufacturer’s protocol. HepG2 cells were seeded in 96 well plates with 10,000 cells per well and cultured as described above. Subsequently, 10 μl of WST-1 reagent was added to each well and absorbance was determined at 450 nm using the Flurostar OPTIMA (BMG Labtech).

### Apoptosis assay

0.1 × 10^6^ HepG2 cells per well were seeded in 12 well plates and cultured as indicated above. Additionally, cells were incubated with camptothecin (2 μM) (Sigma Aldrich) and etoposide (85 μM) (Merck Chemicals) as positive control for apoptosis for 24 h. Apoptosis was measured using the FITC Annexin V Apoptosis Detection Kit (BD Pharmingen™) as described before [18].

### Protein extraction and western blot analyses

0.3 × 10^6^ HepG2 cells per well were seeded in 6 well plates and incubated as described above. Cells were harvested in modified RIPA buffer containing 50 mM TrisHCl pH 7.4; 1% NP-40; 0.25% sodium deoxycholate; 1× Roche complete proteases inhibitor cocktail; 1 mM EDTA; 1 mM sodium orthovanadate, 1 mM sodium fluoride, 5 mM nicotinamide, 5 μM Trichostatin A and 1× Roche Complete protease inhibitor cocktail. Protein amount of cell lysates was determined using BCA protein assay (Pierce, Thermo Scientific). 10 μg or 30 μg protein was separated by SDS-PAGE and semi-dry transferred to nitrocellulose membranes as described before [18]. For detection of NAMPT in the supernatant, 24 μl of samples were used and processed as described above.

### NAD assay

NAD concentrations were measured using the EnzyChrom™ NAD^+^/NADH Assay Kit (E2ND-100, Biotrend) according to manufacturer’s protocol or by HPLC as described before [18].

### Total RNA extraction and realtime qPCR

For total RNA extraction 0.3 × 10^6^ HepG2 cells per well were seeded in 6 well plates. Total RNA was extracted by RNeasy Mini Kit (Qiagen) according to manufacturer’s protocol. 1000 ng of total RNA was transcribed into cDNA by M-MLV Reverse Transcriptase (Invitrogen). Afterwards, Taqman® analyses were performed using the qPCR Master Mix Plus Low ROX (Eurogentec) and the Applied Biosystems 7500 Real Time PCR System. *NAMPT* (forward: 5′-GCA-GAA-GCC-GAG-TTC-AAC-ATC-3′; reverse: 5′- TGC-TTG-TGT-TGG-GTG-GAT-ATT-G-3′; probe: 5′-TGG-CCA-CCG-ACT-CCT-ACA-AGG-TTA-CTC-AC-3′) was normalised to the mean of the housekeeping gene *ß-actin* (forward: 5′-CGA-GCG-CGG-CTA-CAG-CTT-3′; reverse: 5′-CCT-TAA-TGT-CAC-GCA-CGA-TTT-3′; probe: 5′-ACC-ACC-ACG-GCC-GAG-CGG-3′).

### NAMPT enzyme activity

For determination of NAMPT activity 10 × 10^6^ HepG2 cells were seeded in T175 culture flasks and cultured as described above. After lysis in 50 mM 0.1 M sodium phosphate buffer, pH 7.4, 30 μg of protein was added to the reaction buffer and incubated at 37 °C for 2 h. Afterwards the assay using radiolabelled ^14^C–nicotinamide was performed as described before [19].

### Oil red O staining

HepG2 cells were grown in 24-well plates, fixed with phosphate buffered paraformaldehyde solution (Roth), washed with PBS and stained with Oil red O solution (stock solutions: 50 mg Oil red O powder in 100 ml isopropyl) for 15 min at 37 °C. After washing with PBS cells were photographed under the microscope. For quantification Oil red O was dissolved by adding isopropropanol (100%) to the samples. 100 μl of supernatant was added to a 96-well plate and measured at 510 nm using the Flurostar OPTIMA (BMG Labtech) [20].

### Statistical analyses

Statistical analyses were performed with GraphPad Prism® software (5.03). Significance levels were calculated by one-way analysis of variance (ANOVA) followed by Bonferroni post hoc test (*p < 0.05; **p < 0.01; ***p < 0.001 compared to control; ^#^ *p* < 0.05; ^##^ *p* < 0.01; ^###^ *p* < 0.001 compared to palmitate, ^§^ *p* < 0.05; ^§§^ *p* < 0.01; ^§§§^ *p* < 0.001 in a time dependent manner). Data represent means ± SEM of three independent experiments.

## Results

### Palmitate-induced cell death is rescued by oleate

**Fig. 1:**
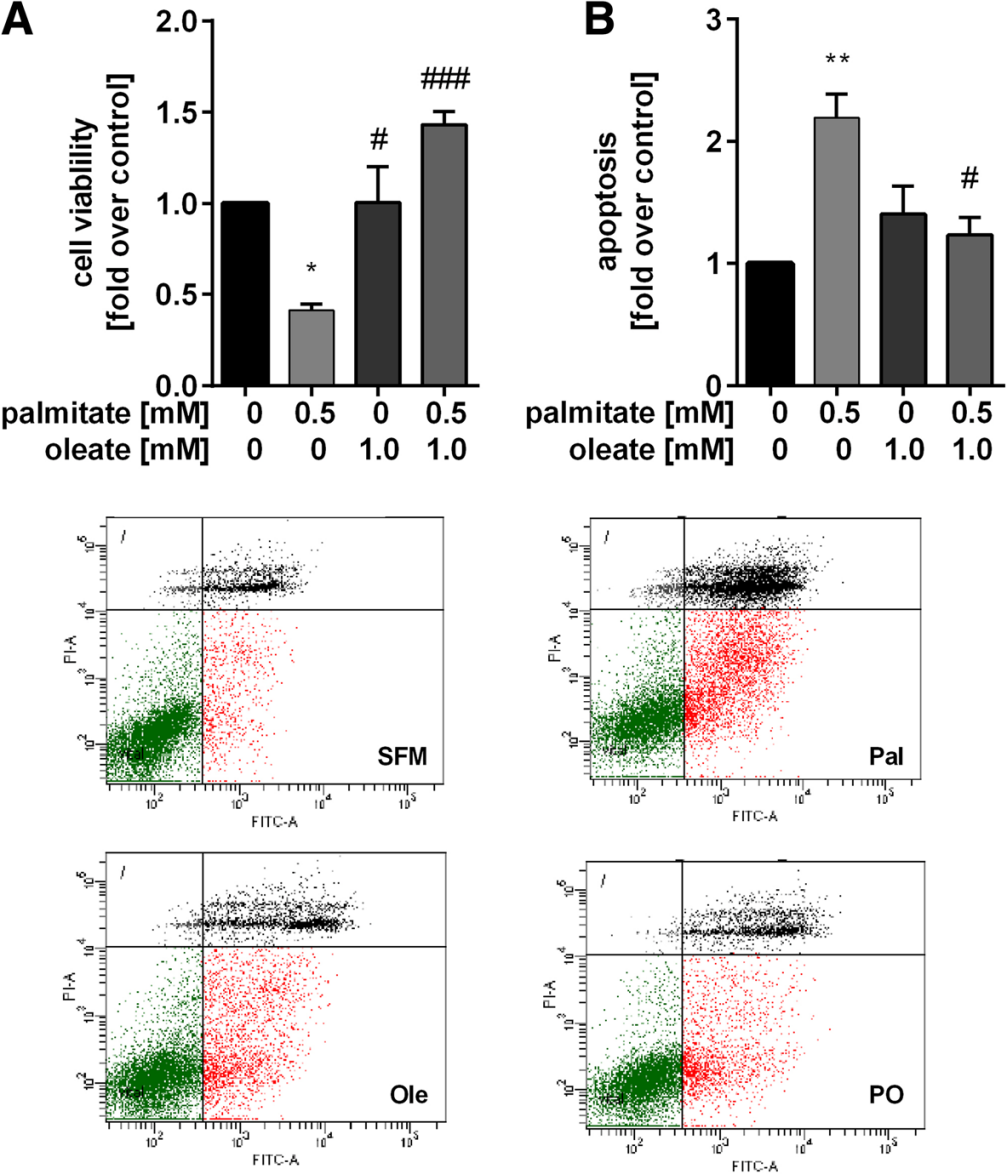
Palmitate-induced cell death is rescued by oleate. HepG2 cells were stimulated with palmitate (0.5 mM), oleate (1 mM) and a combination of both (0.5 mM/1 mM) for 48 h. **a** Cell viability was measured by WST-1 assay. **b** Apoptosis was determined by flow cytometrical counting of Annexin V-FITC and Annexin V-FITC/PI positive stained cells. Data were normalised to control (serum free medium) which was set 1. Representative dot plots of the AnnexinV-FITC/PI staining in HepG2 cells are shown. Data represent three independent experiments performed in triplicates shown as means ± SEM. *p < 0.05, **p < 0.01 compared to control. ^#^ *p* < 0.05, ^###^ *p* < 0.001 compared to palmitate. SFM: serum free medium; Pal: palmitate; Ole: oleate; PO: palmitate/oleate

To investigate the impact of palmitate and oleate on cell survival, HepG2 cells were incubated with palmitate (0.5 mM), oleate (1 mM) and a combination of both (0.5 mM/1 mM). After 4 h of incubation cell viability was unaltered. Palmitate decreased cell viability by 2.0-fold after 24 h (Additional file 2: Figure S2A). Viability further declined by 2.4-fold after 48 h (Fig. 1a). Oleate alone did not influence cell viability, but was able to ameliorate the negative impact of palmitate. Furthermore, oleate was able to normalize the number of apoptotic cells after palmitate-incubation (48 h) from 2.2-fold to control level (Fig. 1b).

### Regulation of NAD salvage pathway by palmitate and oleate

**Fig. 2:**
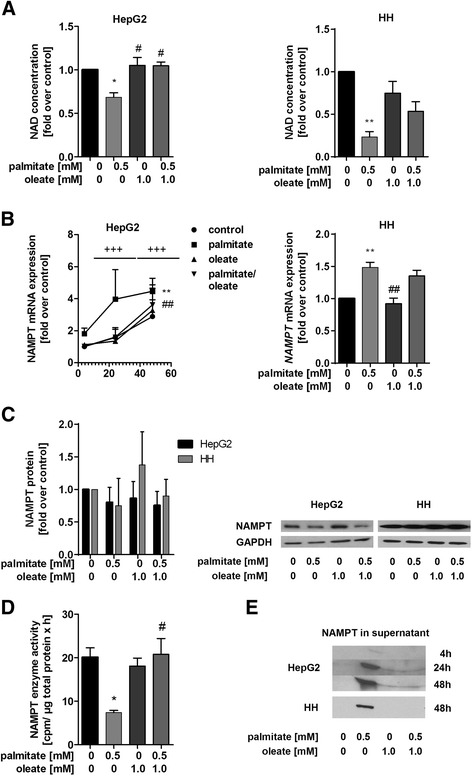
Regulation of NAD salvage pathway by palmitate and oleate. HepG2 cells and primary human hepatocytes were stimulated with palmitate (0.5 mM), oleate (1 mM) and a combination of both (0.5 mM/1 mM) for the indicated time points. **a** Intracellular NAD concentration was measured with EnzyChrom™ NAD^+^/NADH Assay Kit (E2ND-100) or by HPLC (48 h). **b** *NAMPT* mRNA expression was quantified by qPCR and normalised to *β-actin* in HepG2 cells (4 h, 24 h, 48 h) and primary human hepatocytes (48 h). **c** NAMPT protein abundance was measured by Western blot analysis (48 h). GAPDH was used as loading control. One representative Western blot is shown. **d** After 48 h exposure to the indicated free fatty acids NAMPT enzyme activity assay was performed. **e** NAMPT protein in the supernatant of HepG2 cells (4 h, 24 h, 48 h) and primary human hepatocytes (48 h) was determined by Western blot analysis. One representative Western blot is shown. Data were normalised to control (serum free medium) which was set 1. Data represent three independent experiments performed in triplicates shown as means ± SEM. *p < 0.05, **p < 0.01 compared to control. ^#^ *p* < 0.05, ^##^ *p* < 0.01 compared to palmitate. ^§§§^ *p* < 0.001 palmitate in a time dependent manner

NAD is known to play a central role in cellular metabolism as a redox partner or co-substrate for NAD-dependent enzymes. Therefore, intracellular total NAD concentration was measured in HepG2 cells and primary human hepatocytes. Palmitate decreased NAD concentration by 1.2-fold in HepG2 cells and by 3.2-fold in primary human hepatocytes (Fig. 2a) after 48 h. In line with these data, NAMPT activity in HepG2 cells was significantly decreased from 13.8 ± 0.02 cpm/ µg total protein x h to 6.2 ± 1.1 cpm/ µg total protein x h (2.4-fold) after 48 h of palmitate incubation (Fig. 2d). Incubation of HepG2 cells with palmitate led to higher *NAMPT* mRNA expression after 4 h and 24 h which was significantly increased by 5.2-fold after 48 h compared to control. In primary human hepatocytes, palmitate significantly increased *NAMPT* mRNA expression by 1.5-fold after 48 h (Fig. 2b). Interestingly, when we determined the NAMPT protein amount in lysates of HepG2 cells and primary human hepatocytes we found no significant changes in NAMPT protein level after stimulation with palmitate and oleate or its combination (Fig. 2c). Since hepatocytes are a source of extracellular NAMPT (eNAMPT) [21] and eNAMPT has been described as stress-response protein we analysed whether NAMPT was released by the cells and accumulated in the supernatant. After stimulation of HepG2 cells with palmitate NAMPT was secreted into the supernatant already after 4 h, a time point where we detected no cytotoxic effects. Extracellular NAMPT further increased in HepG2 cell supernatants time-dependently after 24 h and 48 h of palmitate treatment. Primary human hepatocytes released NAMPT into the supernatant after stimulation with palmitate after 48 h (Fig. 2e). Oleate alone had no impact on NAD concentration or NAMPT activity and release, but significantly restored palmitate-induced changes on NAMPT mRNA, −activity and NAD levels (Fig. 2a–d).

### NMN does not counteract the palmitate-induced decrease in cell viability

**Fig. 3:**
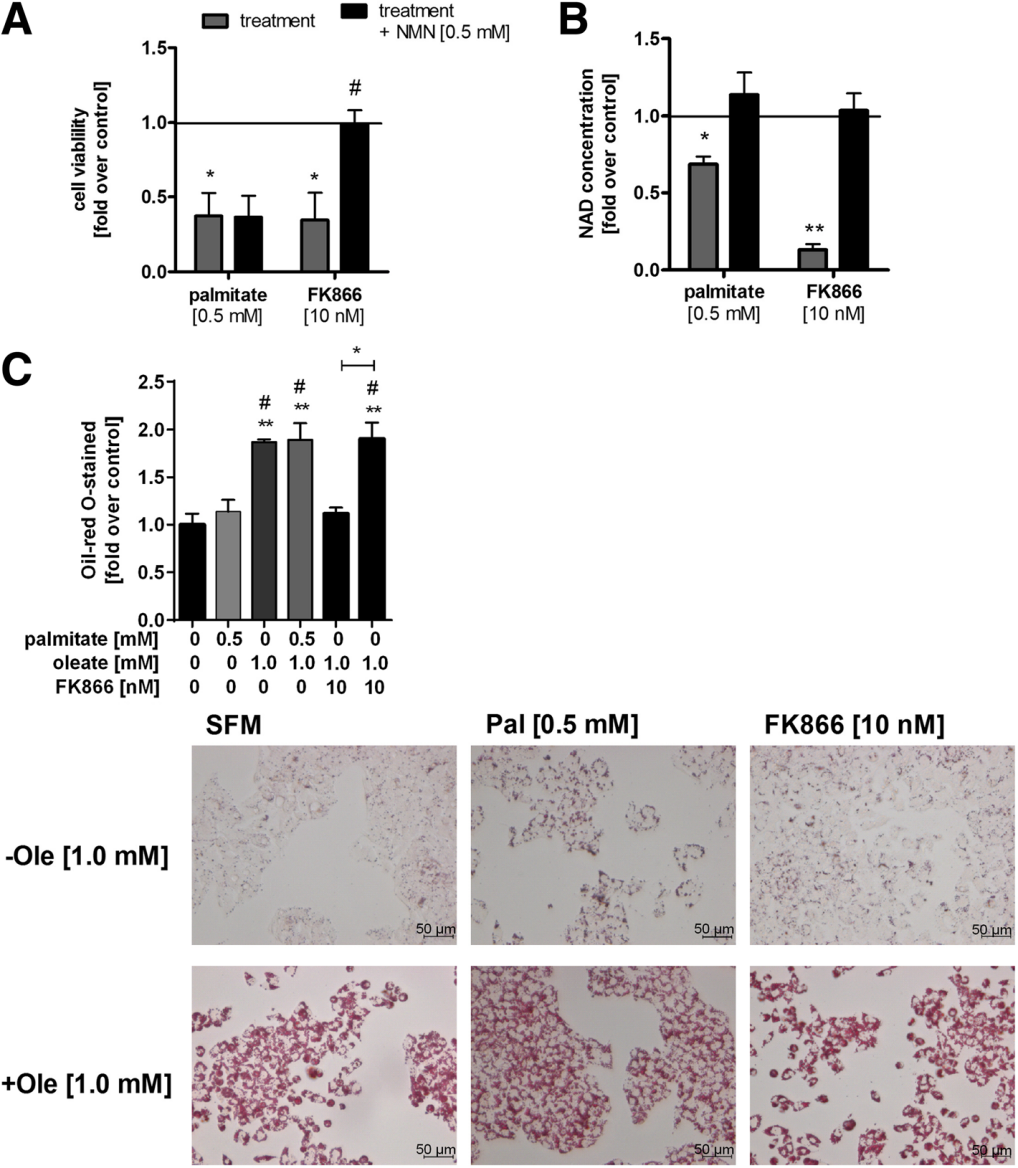
NMN was able to restore reduced NAD concentrations but not cell viability after stimulation with palmitate. NAMPT activity and NAD levels were not involved in hepatic triglyceride storage. **a** HepG2 cells were either stimulated with palmitate (0.5 mM), the specific NAMPT inhibitor FK866 (10 nM), NMN (0.5 mM) or with the indicated combinations for 48 h. Cell viability and **b** NAD concentration were measured using WST-1 assay and EnzyChrom™ NAD^+^/NADH Assay Kit (E2ND-100), respectively. **c** HepG2 cells were stimulated with palmitate (0.5 mM), oleate (1 mM), a combination of both fatty acids (0.5 mM/1 mM), FK866 (10 nM) or with a combination of oleate and FK866. Oil red O staining was performed. Data were normalized to control (serum free medium) which was set 1 and represent three independent experiments performed in triplicates shown as means ± SEM. *p < 0.05, **p < 0.01 compared to control. ^#^ *p* < 0.05, ^##^ *p* < 0.01 compared to equivalent control (palmitate or FK866)

To find out whether or not the decreased NAD levels were responsible for the reduction in cell viability after palmitate treatment, HepG2 cells were co-stimulated with the NAMPT enzyme product NMN. Although intracellular NAD levels were normalized (Fig. 3b), NMN was not able to rescue palmitate-induced cell viability reduction (Fig. 3a). As positive control, we tested whether NMN can improve cell viability of FK866-induced NAD depletion. NMN normalized the reduced cell viability and NAD levels (by 2.8-fold and 3.6-fold, respectively) caused by incubation with the specific NAMPT inhibitor FK866 (Fig. 3a, b).

### A decreased NAMPT activity does not affect lipid accumulation

Lipid content of HepG2 cells after incubation with palmitate, oleate or the combination was assessed. After treatment with palmitate, no significant increase in intracellular lipids was detected, while oleate and a combination of oleate and palmitate treatment resulted in a 1.9-fold increase (Fig. 3c). Since NAMPT expression and NAD concentrations had been shown to be downregulated during the development of fatty liver disease [9] and NAMPT activity has been implicated in the regulation of lipid metabolism in the liver [22], we examined whether a decreased NAMPT activity and NAD levels would lead to higher lipid accumulation. Therefore, HepG2 cells were co-stimulated with FK866 (10 nM). However, FK866 treatment did not lead to alterations in intracellular lipid content in oleate-stimulated hepatocytes (Fig. 3c).

## Discussion

Disturbances in NAMPT and NAMPT-mediated NAD biosynthesis have been reported during the development of NAFLD [9, 23] and NAMPT is implicated in the regulation of lipid metabolism [22, 24]. However, the regulation of NAMPT expression and activity by free fatty acids in hepatocytes is incompletely understood. In our study we, therefore, examined the effect of the two main free fatty acids of human nutrition and adipose tissue, oleate (18:1) and palmitate (16:0) on hepatocyte viability and the regulation of hepatic NAMPT and NAD levels.

We could show that palmitate induces apoptosis in HepG2 cells which can be rescued by oleate. This is in line with several other studies. In INS-1E β-cells palmitate-induced apoptosis was rescued by oleate due to repression of endoplasmic reticulum stress (ER) via eukaryotic Initiation Factor 2α (eIF2α), X-box binding protein 1 (XBP1) and Chop [25]. Stimulation of BiP by oleate or its overexpression restored palmitate-induced ER stress and apoptosis in HepG2 cells [26]. Oleate is able to ameliorate palmitate-induced mitochondrial reactive oxygen species (ROS) generation, concomitant mitochondrial dysfunction and oxidative stress [27, 28].

Since it is known that inhibition of NAMPT-mediated NAD biosynthesis by the NAMPT-specific inhibitor FK866 reduces cell viability and induces apoptosis in hepatocytes [18], we asked whether or not NAMPT is affected by palmitate treatment. Incubation with palmitate led to decreased intracellular NAD levels in HepG2 cells as well as in primary human hepatocytes, which could be explained by a decreased NAMPT activity while intracellular NAMPT protein levels were maintained. How NAMPT activity is influenced by palmitate is still a matter of debate. Our data indicate that NAMPT activity is not associated with SIRT1 activity or ER stress (data not shown). Palmitate could lead to impaired β-oxidation, ROS production and calcium signalling [29]. All of these processes depend on NAD and might play a role in decreased NAMPT activity and NAD levels induced by palmitate. It is known that NAMPT is only active as a dimer [30]. It could be that palmitate increases the monomer to dimer ratio in the cell which decreases NAMPT activity and subsequently NAD levels. Interestingly, *NAMPT* mRNA levels increased and NAMPT accumulated in the supernatant already after 4 h. Our data are in line with earlier findings that resveratrol decreases NAMPT activity and induces *NAMPT* mRNA upregulation as well as NAMPT release in hepatocarcinoma cell lines [31]. Furthermore, in human pancreatic islets glucose leads to an increase of *NAMPT* mRNA expression and accumulation of enzymatically active NAMPT in the supernatant [32]. These findings point to a common mechanism used by different stress signals to induce *NAMPT* mRNA expression and concomitant release of NAMPT. In contrast to our study, palmitate decreases NAMPT release from 3 T3-L1 adipocytes [33], which implicates a cell-type dependent effect of palmitate. Serum NAMPT levels are altered in various diseases, for example in liver diseases [9, 34]. Since it is known that hepatocytes are a source of circulating NAMPT [19], it is of interest to fully understand the regulation of NAMPT release from different cells and organs into the circulation [35]. In adipose tissue, NAMPT secretion is regulated by SIRT1 which deacetylates NAMPT at lysine 53 and thus increases NAMPT activity and secretion. Extracellular NAMPT (eNAMPT) counteracts palmitate-induced apoptosis by activating phosphoinositide 3-kinase (PI3-kinase)-dependent signaling pathways and down regulation of proapoptotic proteins, like B-cell lymphoma 2 (Bcl-2), cytochrome c and caspase 3 in pancreatic β-cells [36]. Furthermore, eNAMPT protects against ER stress-induced apoptosis by activating the interleukin-6/Stat3 pathway via a non-enzymatic function [37].

Inhibition of intracellular NAMPT activity via FK866 leads to increased cell death which can be ameliorated by supplementation with the NAMPT enzyme product NMN [18]. In our study, co-stimulation of hepatocytes with palmitate and NMN led to a normalization of NAD levels. However, cell viability was not ameliorated by NMN co-treatment. In contrast, oleate co-treatment completely reversed the palmitate-mediated cell viability decrease. This could be due to the fact that oleate triggers the conversion of palmitate to neutral triacylglycerides, which directly prevents palmitate from exerting adverse effects like promoting the accumulation of toxic diacylglycerides [27]. Furthermore, it has already been shown that palmitate-induced apoptosis is driven by other cellular processes such as calcium-stimulated mitochondrial activation [28], autophagy [38], ER stress [39] or accumulation of ROS [29] which seemed to be independent of intracellular NAD levels. In animal studies it has been shown that alterations in NAMPT protein levels by pharmacological factors [13, 40] or by gene modification [22] improve hepatic triglycerides but in our study inhibition of NAMPT activity did not lead to a higher accumulation of triglycerides after oleate stimulation. This indicates that NAMPT is not involved in regulating triacylglyceride storage in our cell model. However, another study showed that NAMPT overexpression ameliorated hepatic triglyceride storage after oleate treatment [41].

## Conclusions

We could show that palmitate decreased NAMPT activity and NAD levels in human hepatocytes and NAMPT acts as an early stress-response protein that is upregulated and secreted into the supernatant under lipotoxic stress. However, the pro-apoptotic effects of palmitate seem not to be mediated via intracellular NAD level. Oleate alone had no influence on the tested cellular responses but interestingly ameliorated the adverse impact of palmitate in combined treatment.

## Additional files

Additional file 1: Figure S1. Solvent controls do not influence cell viability as well as intracellular NAMPT and NAD levels. HepG2 cells were stimulated with serum-free medium with and without DMSO (dilution factor: 1:1′000‘000) and serum free medium with 1% free fatty acid -BSA with and without NaOH (dilution 1:200). Neither DMSO nor NaOH altered cell viability measured by WST-1 assay (A), intracellular NAD levels measured by HPLC (B), NAMPT mRNA expression or protein abundance measured by qPCR or Western blot analysis (C,D), respectively, enzyme activity (E) and lipid accumulation stained with Oil-red O (F). Data were normalised to control (serum free medium) which was set 1. Data represent two or three independent experiments shown as means ± SEM. FFA: free fatty acid. (JPEG 691 kb)

Additional file 2: Figure S2. Palmitate decreases cell viability after 24 h. After stimulation of HepG2 cells with palmitate and oleate for 4 h and 24 h at the indicated concentrations, cell viability was measured via WST-1 assay. Data were referred to control (serum free medium) and represent three independent experiments performed in triplicates shown as means ± SEM. ***p < 0.05 compared to control. (JPEG 51 kb)

## Abbreviations

Bcl2: B cell lymphoma 2
eIF2α: eukaryotic Initiation Factor 2α
eNAMPT: Extracellular NAMPT
ER: Endoplasmic reticulum
NAD: Nicotinamide adenine dinucleotide (NAD)
NAFLD: Non-alcoholic fatty liver (NAFLD)
NAMPT: Nicotinamide phosphoribosyltransferase
NMN: Nicotinamide mononucleotide
PBEF: Pre-B cell colony-enhancing factor (PBEF)
PI3K: Phosphoinositide 3-kinase
ROS: Reactive oxygen species
XBP1: X-box binding protein 1
