# Supplementary figures and images for: Oleate ameliorates palmitate-induced reduction of NAMPT activity and NAD levels in primary human hepatocytes and hepatocarcinoma cells
Source: Lipids Health Dis. 2017 Oct 3;16:191. doi: 10.1186/s12944-017-0583-6 (PMC5627432; doi:10.1186/s12944-017-0583-6)

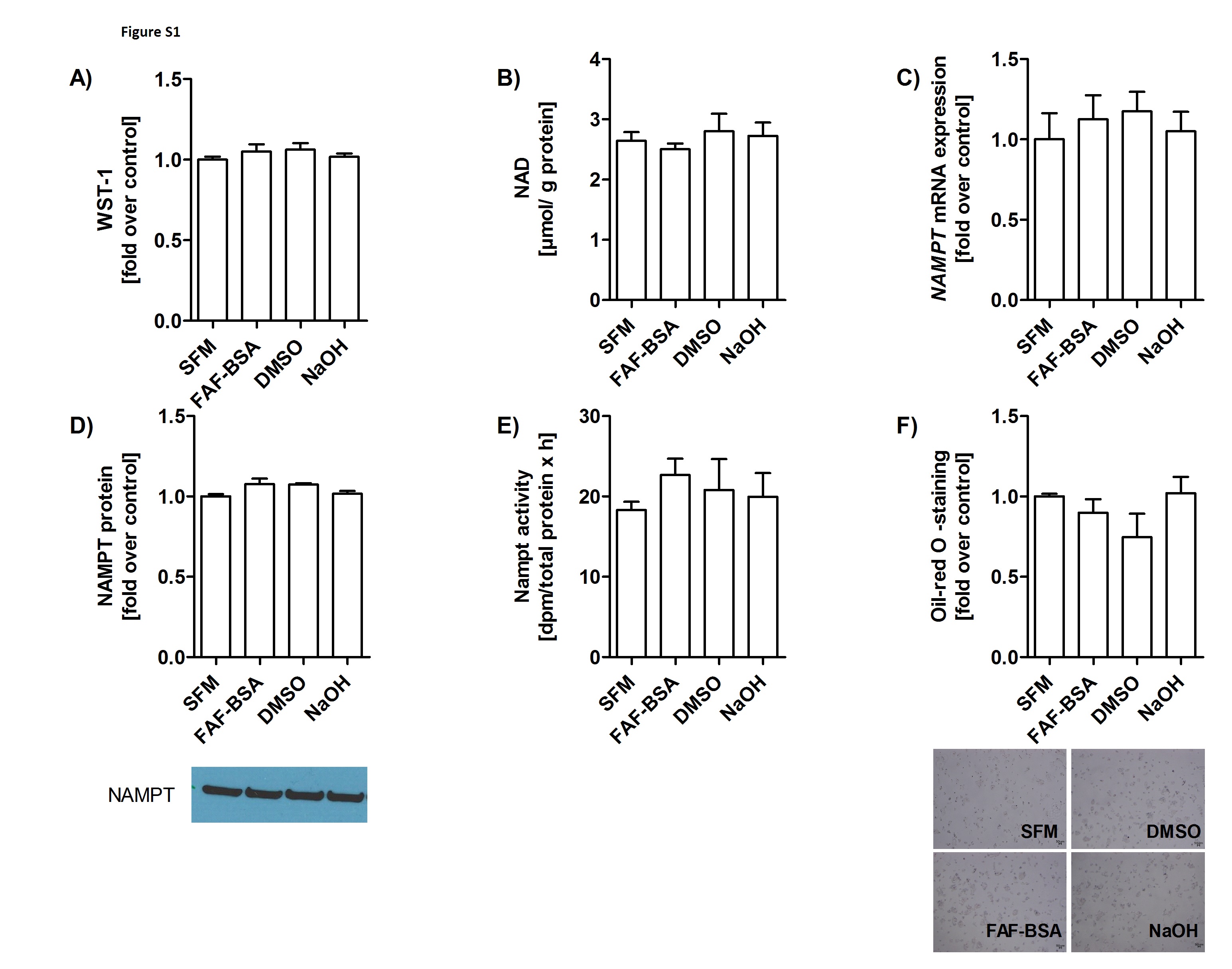

Supplement: Supplementary file 1 — Solvent controls do not influence cell viability as well as intracellular NAMPT and NAD levels. HepG2 cells were stimulated with serum-free medium with and without DMSO (dilution factor: 1:1′000‘000) and serum free medium with 1% free fatty acid -BSA with and without NaOH (dilution 1:200). Neither DMSO nor NaOH altered cell viability measured by WST-1 assay (A), intracellular NAD levels measured by HPLC (B), NAMPT mRNA expression or protein abundance measured by qPCR or Western blot analysis (C,D), respectively, enzyme activity (E) and lipid accumulation stained with Oil-red O (F). Data were normalised to control (serum free medium) which was set 1. Data represent two or three independent experiments shown as means ± SEM. FFA: free fatty acid. (JPEG 691 kb) [file 12944_2017_583_MOESM1_ESM.jpg]

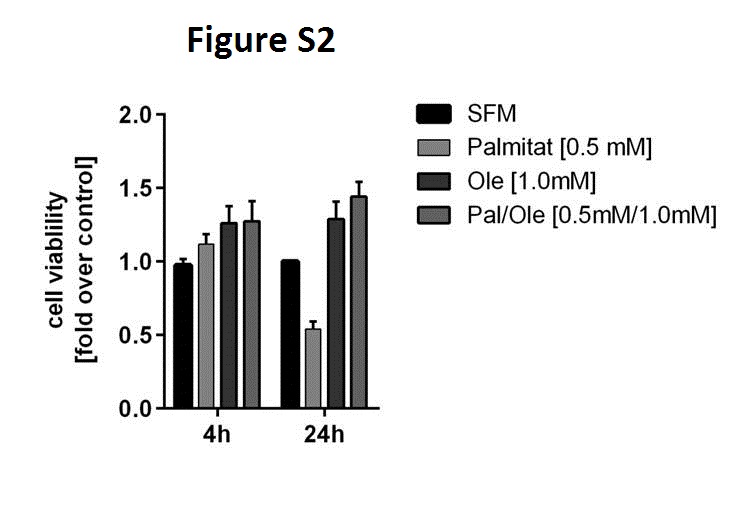

Supplement: Supplementary file 2 — Palmitate decreases cell viability after 24 h. After stimulation of HepG2 cells with palmitate and oleate for 4 h and 24 h at the indicated concentrations, cell viability was measured via WST-1 assay. Data were referred to control (serum free medium) and represent three independent experiments performed in triplicates shown as means ± SEM. ***p < 0.05 compared to control. (JPEG 51 kb) [file 12944_2017_583_MOESM2_ESM.jpg]
